# Correlations between Self-Reported Cooking Confidence and Creativity and Use of Convenience Cooking Products in an Australian Cohort

**DOI:** 10.3390/nu13051724

**Published:** 2021-05-19

**Authors:** Natasha Brasington, Patrice Jones, Tamara Bucher, Emma L. Beckett

**Affiliations:** 1 School of Environmental and Life Sciences, The University of Newcastle, Ourimbah, NSW 2258, Australia; Natasha.Brasington@uon.edu.au (N.B.); Patrice.Jones@newcastle.edu.au (P.J.); Tamara.Bucher@newcastle.edu.au (T.B.); 2 Hunter Medical Research Institute, New Lambton Heights, NSW 2305, Australia; 3 Priority Research Centre for Physical Activity and Nutrition, The University of Newcastle, Callaghan, NSW 2308, Australia

**Keywords:** convenience cooking products, cooking confidence, cooking creativity, food behavior

## Abstract

Background: Most Australians do not meet vegetable intake recommendations. Vegetables are most often consumed in evening meals. However, they often require preparation and therefore cooking skills. Convenience cooking products such as meal bases/concentrates and ready-made sauces are increasingly common and popular and may help address the barriers to vegetable consumption in terms of cost and time. These products also typically provide recipes, which include vegetables, and as such, may help address the barriers of cooking skills, confidence, and creativity. However, the relationships between the use of these products, cooking confidence, and cooking creativity remain unknown. Methods: Australian adults were surveyed (snowball recruitment, n = 842) on their use of convenience cooking products (meal bases/recipe concentrates, simmer sauces, marinades, and other cooking sauces), cooking confidence (7 item scale) and creativity (6 item scale), and demographic information. Results: Overall, 63.2% of participants reported using convenience cooking products. *Those using these products had lower mean cooking skills confidence and creativity scores than those who did not, in all product categories assessed. Among users, those who reported "always" following the recipes provided had lower mean cooking confidence and creativity scores than those who followed the recipes less regularly.* Conclusions: Therefore, improving the vegetable content of recipes provided with these products may be a tool to increase vegetable intake by users with lower cooking skills (confidence and creativity). This may complement traditional approaches such as education in improving vegetable intake.

## 1. Introduction

In 2018, the Australian National Health Survey found that 95% of the Australian population did not consume the recommended five servings (~375 g) of vegetables per day [1]. Over the 10 years from 2007 to 2018, there have been no significant changes in these statistics [1]. New strategies are needed to improve vegetable intake, as diets high in vegetables support overall health. In 2017, poor diet was the fifth leading risk factor for mortality worldwide [2]. Studies have shown that negative dietary impacts have increased [3], as have the associated healthcare costs [4]. Diet-related diseases such as obesity, diabetes, cardiovascular diseases, cancer, osteoporosis, and dental diseases are rapidly impacting the health of the Australian population [5,6,7,8].

Higher consumption of food prepared in the home is associated with improved diet quality [9]. However, potential barriers to home cooking include lack of time (actual and perceived), perceived affordability of healthy products compared to convenience foods, longer working hours, and accessibility to and perceived effort of preparing fresh food [10,11,12,13,14]. Individuals also may not have the necessary skills and confidence levels to prepare healthful meals [10,11,12,13,14]. Knowledge of nutrition and cooking skills can all influence an individual’s food choices [15,16]. Cooking is a valuable life skill linked to improved diet quality, including increased consumption of fruits and vegetables, and an improved recognition of healthful foods [17,18]. In a cross-sectional survey in Australia, more confidence with food and cooking skills was associated with higher diet quality in men [19]. Therefore, programs to increase cooking skills have been increasingly utilized as a preventive measure to address diet-related diseases, including obesity, by improving dietary outcomes [20]. Despite this, vegetable intake in Australia remains low, and diet-related diseases, overweight, and obesity remain growing health concerns [21].

When individuals lack cooking skills, they become susceptible to messages about ease of preparation and taste [22]. As such, the use of convenience and processed food has increased, reducing the need for cooking skills [23]. The term “convenience cooking product” refers to products that provide a base for cooking meals that lowers the cooking time and preparation time. Convenience cooking products, in particular meal bases and recipe bases, have become common in the average Australian household [24]. Meal and recipe bases tend to be in a liquid or powder form and have a suggested recipe provided on the pack (also referred to as a back-of-pack recipe). Cooking sauces (e.g., pasta sauces and simmer sauces) are typically sold in jars, and marinades in jars or sachets. Convenience cooking products are often perceived as non-nutritious, i.e., energy-dense, high in fats and carbohydrates, and lacking in essential micronutrients [25,26,27]. However, the back-of-pack recipes on these products suggest vegetables, protein and/or grains to be added by the consumer during the preparation process. Consequently, when used correctly, i.e., if consumers follow the back-of-pack recipe instructions, these products may potentially help to increase the number of vegetables servings consumed. Convenience products, therefore, may have the potential to improve diet quality in those who are time-poor or have low levels of cooking skills.

However, there are no data on the relationship between the use of convenience cooking products and cooking skills. Therefore, we conducted a cross-sectional survey study using scales of cooking skill confidence and creativity and examined this in the context of the use of convenience home cooking products (meal/recipe bases, simmer sauces, marinades, and other cooking sauces). Information collected included categories of products used, frequency of use, and frequency of following the recipes provided with the products. This information could be useful when considering the roles of these products in a modern diet and in designing future interventions.

## 2. Materials and Methods

### 2.1. Study Design and Recruitment

This cross-sectional survey was granted ethical approval by The Human Research Ethics Committee of the University of Newcastle (Reference No. H-2020-0119). A snowball recruitment method was used to recruit a convenience sample. The survey was created and administered online through Qualtrics (SAP, USA). Questions were organized into thematic blocks based on cooking skills (confidence and creativity), meal/recipe base usage habits, and demographics. The survey was advertised online for ~7 weeks (09/06/2020–29/07/2020) on investigators’ and the University of Newcastle’s social media pages, such as Facebook, Twitter, and Instagram. Participants were included in the study if they were living in Australia and were over 18 years of age. Proficient English comprehension skills were a practical requirement for participation.

### 2.2. Frequency of Use and Adherence to Recipes 

Participants were asked about their current usage of convenience cooking products. Participants who reported using meal and recipe bases (or concentrates), simmer sauces, marinades, or other cooking sauces were classified as “users”, of that product category and “non-users” if they did not use these products. Product users were then asked what brands of the products they currently use, from a list of common brands in Australian supermarkets. A free answer option was also provided. Users were asked how often they use products, selecting from multiple times a week, once a week, once a month, or less than once a month. These categories were collapsed into 3 groups (>weekly, weekly, and <weekly) for analysis. Users were then asked if they follow the recipe provided on the back of the pack and how often (always, sometimes, or never).

### 2.3. Cooking Confidence Scores and Cooking Creativity Scores 

The cooking confidence and creativity scales included in this questionnaire were based on the cooking identity and food creativity scales used in a previously validated questionnaire [23], with minor modifications. The cooking identity scale was used as a measure of cooking confidence, as it contains items such as "others view me as a good cook". A minor modification to this scale was made to remove "I can time different elements of a dish to come together on time" and replace it with "I don’t consider myself to be a good cook" to serve as an additional attention check. The food creativity scale was used as a measure of cooking creativity and included statements such as "I am good at coming up with new and different recipe ideas" and "I don’t have much of an imagination about things to cook". The complete scales are included in the Supplementary Materials (Supplementary Methods). Cronbach’s Alpha was calculated to assess the internal validity of the scales. The statements were rated on a 5-point rating scale that ranged from 1 (strongly disagree) to 5 (strongly agree). These ratings were summed to calculate a cooking confidence (maximum score 35) and cooking creativity score (maximum score 30) for each participant. Reversed style questions of cooking confidence and creativity were used as an attention check to test if participants were reading the questions carefully.

### 2.4. Statistical Analysis 

Data were analyzed using JMP (Pro 14; SAS Institute Inc., Cary, NC, USA). Both categorical and continuous data were used. The statistically significant threshold was a *p*-value of <0.05. Contingency tables (Pearson χ^2^) were used to assess the relationships between categorical variables. Standard least squares regression was used to compare adjusted least squares means by category (adjusted for age, sex, income, education, and working hours).

## 3. Results

### 3.1. Demographics

A total of 964 participants responded to the survey. Ninety-nine participants were excluded for incomplete responses, and a further 23 participants were excluded for completing the survey in less than half the median completion time (<244 s). Overall, there was a total of 842 participants in the final sample, all of which passed the attention check.

Respondent ages ranged from 18–80 years (median; 41 years, standard deviation: 12.1 years). The majority (77.7%) of respondents were female and the majority had a university level education (Table 1). The majority had household incomes above AU$75,000 per year and reported working full-time (>30 h per week; Table 1). 

### 3.2. Convenience Cooking Products—The Proportion of Product Users, Frequency of Use, and Frequency of Recipe Adherence

Overall, 63.2% of participants reported using one or more convenience cooking products. The category of convenience cooking products used by the largest proportion of participants was “other cooking sauces” with 338 participants reporting using this product category, followed by “meal/recipe bases” with 309 participants (Table 2). Of those who used convenience cooking products, meal/recipe bases were the most frequently used, with 22.2% of those who used them reporting using them more than once a week, and 44.9% reporting using them weekly (Table 2). In every product category, the largest proportion of users reported that they sometimes follow the back-of-pack recipes provided with these products, with the majority reporting adherence “sometimes” or “never” (Table 2). 

### 3.3. Cooking Confidence and Cooking Creativity by Use of Convenience Cooking Products

The internal reliability of the cooking confidence and creativity scales was high, with Cronbach’s Alpha scores of 0.90 and 0.87, respectively. The cooking confidence scores ranged from 8 to 35 (possible range 7–35), with a sample mean of 27.0 (standard deviation 5.1). The cooking creativity scores ranged from 6 to 30 (possible range 6–30), with a sample mean of 18.1 (standard deviation 5.2). 

Mean cooking confidence scores were lower among users of meal/recipe bases, simmer sauces, and other cooking sauces compared to non-users of these products (Table 3). These results remained significant when analyses were adjusted for age, sex, income, education, and work hours. There was no difference in cooking confidence scores between users and non-users of marinades, both in unadjusted and unadjusted analyses (Table 3). 

In all convenience cooking product categories, product users had lower mean cooking creativity scores compared to non-users of these products (Table 4). These results remained significant when analyses were adjusted for age, sex, income, education, and work hours (Table 4).

### 3.4. Cooking Confidence and Cooking Creativity by Recipe Adherence Habits 

In all products, the cooking confidence scores were lower among those who used the products and always followed the recipes provided, compared to those who sometimes or never followed the recipes provided (Table 5). These results remained significant when analyses were adjusted for age, sex, income, education, and work hours (Table 5).

In all product categories assessed, the cooking creativity scores were lower among those who always followed the recipes provided, compared to those who sometimes or never followed the recipes provided (Table 6). These results remained significant when analyses were adjusted for age, sex, income, education, and work hours (Table 6).

### 3.5. Cooking Confidence and Cooking Creativity by Frequency of Use

Neither cooking confidence (Table 7) nor creativity (Table 8) varied by frequency of product use, for any product category assessed. These results did not vary when analyses were adjusted for age, sex, income, education, and work hours.

## 4. Discussion

This study is the first to examine the use of convenience cooking products, cooking confidence, and cooking creativity. Use of convenience cooking products across all categories was common in this cohort. Cooking confidence scores were low among users of all categories of convenience cooking products compared to non-users of the products. The presented findings demonstrated that those who reported always following provided back-of-pack recipes had lower cooking confidence and creativity scores compared to those who reported sometimes or never following recipes. However, among users, frequency of use was not associated with changes in either cooking confidence or creativity scores. This may be due to the frequency categories used or the overlap in the multiple categories of products assessed.

The differences in the cooking confidence and creativity scale scores between groups were statistically significant but numerically small. However, it is important to remember that the indices used are unit-less, and the values are arbitrary. The data obtained here can be compared to the data of Lavelle et al. [23], as the cooking creativity scale used here was identical to their food creativity scale, and the cooking confidence scale used here contained only one modified question. For the cooking confidence scale, the scores for users of convenience cooking products obtained here reflect those in the lower two quartiles of the representative Australian sample studied in Lavelle et al. [23]. Comparing the scores for the two extremes of recipe-following shows that those who always follow the recipes had scores reflective of the lowest quartile of confidence in the representative sample, and those who never follow the recipes had scores that reflect the second highest quartile of the representative sample. For the cooking creativity scale, the scores for users of convenience cooking products obtained here reflect those in the lowest of the representative Australian sample studied in Lavelle et al. [23], and the scores for non-users reflect those in the middle quartiles. Comparing the scores for the two extremes of recipe-following shows that those who always follow the recipes had scores reflective of the lowest quartile of creativity in the representative sample, and those who never follow the recipes had scores that reflect the second lowest quartile of the representative sample.

Cooking skills have been categorized as a necessary skill. These skills are associated with self-reliability and a basic knowledge of nutrition, and influence individuals’ dietary behaviors and subsequent overall health [28]. Research suggests that individuals lacking cooking skills tend to consume higher amounts of pre-prepared convenience foods, commonly of lower nutritional value compared to meals prepared at home [29,30,31,32,33,34]. There has been a decline in common cooking skills in recent years that could be attributed to many factors [28]. Here, use of convenience cooking products was associated with lower cooking confidence and creativity scores, when compared to non-users of these products. Among users, the frequency of use did not appear to be related to cooking confidence and creativity, with no significant differences in the cooking confidence and creativity scores by the frequency of product use. Interestingly, users who reported always following provided back-of-pack recipes had lower cooking confidence and cooking creativity scores compared to users who followed recipes less regularly (either "sometimes" or "never"). 

These findings suggest these products, and the recipes provided, are more likely to be utilized by individuals with lower cooking skills. As such, if coupled with other improvements, such as the Australian government’s Healthy Food Partnerships reformulation targets [35], these products, designed to include sufficient vegetables in their back-of-pack recipes, may be a tool to tackle poor-quality diets among individuals with poor cooking skills. This could be especially useful to encourage people away from higher-energy and pre-prepared convenience and discretionary food choices. Convenience cooking products are typically used to prepare evening meals, where the majority of vegetables are typically consumed [36,37]. However, there are currently no compiled independent data describing the vegetable content of these products, and recipes are likely to vary widely.

Cooking skills contribute to overall diet quality and vegetable consumption [38], with a link between low cooking skills and health outcomes in a variety of socio-economic groups [22]. As such, the results presented here regarding differing levels of confidence and creativity among users compared to non-users, and among users by frequency of recipe adherence, may have implications for health outcomes. A population-based longitudinal study of 1710 participants aged 18 to 23 years based in Minnesota USA, found that young adults who reported frequent food preparation reported less frequency of fast-food use [31,35,36,37,38]. These findings suggest that higher cooking skills contribute to a more balanced, healthier diet. However, convenience cooking products may allow increased home meal preparation for those with lower skills and confidence.

Cross-sectional studies have suggested that cooking at home is associated with healthier diet choices and higher consumption of vegetables when compared to eating out from home, which is associated with increased consumption of ready-to-eat meals and calorie-dense convenient foods [9,36,37,39]. There has been limited research on cooking skills and their effect on the Australian diet. Cross-sectional questionnaire studies have reported that older participants, females, and those with higher levels of education are linked to having higher levels of cooking skills in comparison to those who are younger, male or, have a lower level of education. It was also reported that higher diet quality scores were associated with higher cooking and food skills confidence and that participants with such higher scores were more likely to consume fewer takeaway foods [23,31,38,40]. This supports the findings of an Australian study of main household dietary decision makers, which found that the people in the lowest confidence group were significantly more likely to report convenience ingredient use [41]. Similarly, semi-structured interviews with 11 women in Australia and New Zealand suggested that the greater the perceived time pressures, the greater the likelihood of seeking convenience options [42]. Therefore, if properly designed, convenience cooking products could be used as a tool to encourage more home cooking by those with low levels of skills and confidence, and particularly by those also encountering other barriers such as time or cost.

This study is the first to show the association between cooking creativity and confidence scores and the use of convenience cooking products. However, these results must be considered in the context of the limitations of the study design. As this study was highly novel, a convenience recruitment technique of snowball sampling was used in the first instance, leading to a study sample biased towards highly educated women. This is a common limitation of snowball recruitment and voluntary surveys in general [9,43]. However, women are far more likely to cook than men in Australia, with women reporting spending almost twice the amount of time preparing meals as men [39,44]. The distribution of education levels was unbalanced; 71% of the cohort completed a tertiary level of education, while in 2019 it was reported that only 28.2% of Australian adults had tertiary qualifications [40,45]. Different levels of education can influence food choices, with research suggesting that higher levels of education are associated with a healthier lifestyle and lower risk of diet-related diseases [41,46]. As such, results may be more pronounced in a less educated cohort. Furthermore, the data were collected in 2020 during the COVID-19 pandemic, which may have impacted cooking habits at the time. It should also be considered that no description or example of the product categories was provided, which may have led to differences in interpretation. Lastly, there was no available literature for comparative analysis with limited knowledge and literature on meal/recipe bases. Despite these potential limitations, the mean scores for this sample were similar to those obtained by Lavelle et al. [23] in a more representative population, using the original scales that were modified for use in this study. This study, with limitations considered, represents an important first step regarding collating the first research within this field of work.

## 5. Conclusions

Research into convenience cooking products is important, as these products are becoming more common in the Australian household, and cooking skills are declining. Given the association between use of these products and frequency of recipe-following and cooking confidence and creativity, further investigations are warranted into the relationships and the potential for these products to be used as tool to improve vegetable intake and diet quality. This may complement traditional approaches such as education in improving vegetable intake and product reformulation, while addressing barriers to vegetable intake such as skills, confidence, convenience, cost, and time.

## Figures and Tables

**Table 1:** Demographics (n = 842).

Confounder	N	% of Subjects
**Sex**		
Male	171	20.3
Female	654	77.7
Others	17	2.0
**Income**		
<$20,000 per year	25	3.0
$20,000 to $49,999 per year	88	10.5
$50,000 to $74,999 per year	84	10.0
$75,000 to $149,999 per year	303	36.0
>$150,000 per year	235	27.9
Declined to respond or did not know	107	12.7
**Working hours**		
<15 h	173	20.5
15–30 h	151	17.9
30–50 h	455	54.9
50+ h	56	6.7
**Education**		
Below year 12 or equivalent	28	3.3
Year 12 or equivalent	89	10.6
Technical diploma	116	13.8
Bachelor’s degree	290	34.4
Postgraduate degree	314	37.3

**Table 2:** Distribution of convenience cooking product use, frequency of use, and frequency of recipe adherence.

	n (% Total)		*Frequency of Use* n (% Users)			*Frequency of Recipe Adherence* n (% Users)		
Cooking Product	Non-Users	Users	>Weekly	Weekly	<Weekly	Always	Sometimes	Never
Meal/recipe bases	533 (63.3)	309 (36.7)	41 (22.2)	83 (44.9)	61 (33.0)	77 (24.9)	131 (42.4)	101 (32.7)
Marinades	605 (71.8)	237 (28.1)	25 (18.1)	59 (42.7)	54 (39.1)	52 (21.9)	104 (43.9)	81 (34.2)
Simmer sauces	643 (76.4)	199 (23.6)	24 (18.6)	61 (47.3)	44 (34.1)	63 (31.7)	61 (30.6)	75 (37.7)
Other cooking sauces	504 (59.8)	338 (40.1)	42 (21.1)	85 (42.7)	72 (36.2)	77 (22.8)	155 (45.9)	106 (31.4)

**Table 3:** Cooking confidence score by use of convenience cooking products.

	Unadjusted			Adjusted *		
	Mean (95% CI)		*p*	Mean (95% CI)		*p*
Cooking Product	User	Non-User		User	Non-User	
Meal/recipe bases	25.7 (25.2–26.3)	27.7 (27.3–28.2)	<0.0001	26.1 (24.7–27.5)	28.0 (26.7–29.4)	<0.0001
Marinades	26.5 (25.8–27.2)	27.2 (26.8–27.6)	0.07	26.8 (25.4–28.3)	27.4 (26.1–28.8)	0.1
Simmer sauces	25.3 (24.6–26.0)	27.5 (27.2–27.9)	<0.0001	25.7 (24.2–27.1)	27.8 (26.4–29.1)	<0.0001
Other cooking sauces	25.6 (25.1–26.2)	27.9 (27.5–28.4)	<0.0001	26.1 (24.7–27.4)	28.5 (27.1–29.9)	<0.0001

* Adjusted for age, sex, income, education, and working hours.

**Table 4:** Cooking creativity score by use of convenience cooking products.

	Unadjusted			Adjusted *		
Cooking Product	Mean (95% CI)		*p*	Mean (95% CI)		*p*
	User	Non-User		User	Non-User	
Meal/recipe bases	16.6 (16.0–17.1)	19.0 (18.5–19.4)	<0.0001	17.0 (15.6–18.4)	19.4 (18.0–20.7)	<0.0001
Marinades	17.1 (16.5–17.8)	18.4 (18.0–18.9)	0.0009	17.7 (16.2–19.1)	18.8 (17.5–20.3)	0.003
Simmer sauces	16.6 (15.9–17.3)	18.5 (18.1–18.9)	<0.0001	17.1 (15.6–18.6)	18.9 (17.6–20.3)	<0.0001
Other cooking sauces	16.4 (15.9–17.0)	19.2 (18.7–19.6)	<0.0001	17.1 (15.7–18.4)	19.9 (18.6–21.3)	<0.0001

* Adjusted for age, sex, income, education, and working hours.

**Table 5:** Cooking confidence score by recipe-following habits among users of convenience cooking products.

	Unadjusted				Adjusted *			
Cooking Product	Mean (95% CI)			*p*	Mean (95% CI)			*p*
	Always	Sometimes	Never		Always	Sometimes	Never	
Meal/recipe bases	24.7 ^a^ (23.8–25.6)	27.0 ^b^ (26.2–27.7)	26.9 ^b^ (26.3–27.6)	0.0001	25.9 ^a^ (24.1–27.7)	27.8 ^b^ (26.1–29.4)	28.0 ^b^ (26.3–29.6)	0.001
Marinades	24.5 ^a^ (23.6–25.4)	26.8 ^b^ (26.0–27.6)	26.9 ^b^ (26.3–27.5)	<0.0001	25.8 ^a^ (24.0–27.6)	27.7 ^b^ (26.0–29.3)	28.0 ^b^ (26.3–29.7)	0.0007
Simmer sauces	24.6 ^a^ (23.6–25.5)	26.7 ^b^ (26.0–27.5)	26.5 ^b^ (25.9–27.2)	0.0005	25.8 ^a^ (24.0–27.6)	27.6 ^b^ (26.0–29.3)	27.7 ^b^ (26.0–29.4)	0.003
Other cooking sauces	24.8 ^a^ (23.9–25.7)	27.1 ^b^ (26.3–27.8)	27.1 ^b^ (26.5–27.8)	<0.0001	25.3 ^a^ (23.7–26.8)	27.1 ^b^ (25.7–28.5)	27.3 ^b^ (25.9–28.5	0.001

* Adjusted for age, sex, income, education, and working hours. Values in the same row (within an analysis) denoted with the same letter are not significantly different from each other (*p* > 0.05).

**Table 6:** Cooking creativity score by use of convenience cooking products.

	Unadjusted				Adjusted *			
Cooking Product	Mean (95% CI)			*p*	Mean (95% CI)			*p*
	Always	Sometimes	Never		Always	Sometimes	Never	
Meal/recipe bases	14.9 ^a^ (14.1–15.8)	17.5 ^b^ (16.8–18.3)	18.1 ^b^ (17.5–18.7)	<0.0001	15.9 ^a^ (14.2–17.7)	18.2 ^b^ (17.4–20.6)	19.0 ^b^ (17.4–20.6)	<0.0001
Marinades	14.8 ^a^ (13.9–15.6)	18.1 ^b^ (17.5–18.7)	17.4 ^b^ (16.6–18.2)	<0.0001	15.8 ^a^ (14.1–17.6)	18.1 ^b^ (16.5–19.7)	19.0 ^b^ (17.4–20.6)	<0.0001
Simmer sauces	14.8 ^a^ (13.9–15.6)	17.4 ^b^ (16.6–18.1)	18.0 ^b^ (17.4 –18.7)	<0.0001	15.8 ^a^ (14.1–17.6)	18.1 ^b^ (16.5–19.7)	18.9 ^b^ (17.3–20.6)	<0.0001
Other cooking sauces	15.0 ^a^ (14.2–15.9)	17.7 ^b^ (16.9–18.4)	18.3 ^b^ (17.7–18.9)	<0.0001	16.3 ^a^ (14.6–18.0)	18.6 ^b^ (17.0–20.2)	19.4 ^b^ (17.8–21.0)	<0.0001

* Adjusted for age, sex, income, education, and working hours. Values in the same row (within an analysis) denoted with the same letter are not significantly different from each other (*p* > 0.05).

**Table 7:** Cooking confidence score by frequency of use—users of convenience cooking products only.

	Unadjusted				Adjusted *			
Cooking Product	Mean (95% CI)			*p*	Mean (95% CI)			*p*
	>Weekly	Weekly	<Weekly		>Weekly	Weekly	<Weekly	
Meal/recipe bases	27.0 (26.0–28.0)	26.5 (25.8–27.1)	26.5 (25.7–27.2)	0.7	27.4 (25.6–29.3)	27.2 (25.5–28.9)	27.1 (25.4–28.9)	0.9
Marinades	27.0 (25.9–28.0)	26.6 (25.9–27.3)	26.7 (25.9–27.5)	0.9	27.3 (25.4–29.2)	27.3 (25.5–29.0)	27.3 (25.5–29.1)	1.0
Simmer sauces	26.5 (25.4–27.5)	26.2 (25.5–26.9)	26.2 (25.3–27.0)	0.9	27.0 (25.2–28.9)	27.0 (25.3–28.7)	27.0 (25.2–28.7)	1.0
Other cooking sauces	27.0 (26.0–28.0)	26.5 (25.8–27.1)	26.6 (25.9–27.4)	0.7	27.7 (25.9–29.5)	27.5 (25.8–29.1)	27.6 (25.9–29.3)	0.9

* Adjusted for age, sex, income, education, and working hours.

**Table 8:** Cooking creativity score by frequency of use—users of convenience cooking products only.

	Unadjusted				Adjusted *			
Cooking Product	Mean (95% CI)			*p*	Mean (95% CI)			*p*
	>Weekly	Weekly	<Weekly		>Weekly	Weekly	<Weekly	
Meal/recipe bases	18.2 (17.2–19.2)	17.7 (17.0–18.3)	18.0 (17.2–18.7)	0.7	18.7 (16.9–20.6)	18.5 (16.8–20.2)	18.5 (16.8–20.3)	0.9
Marinades	18.0 (16.9–19.0)	17.7 (17.0–18.4)	18.0 (17.0–18.4)	0.8	18.6 (16.7–20.4)	18.5 (16.7–20.1)	18.6 (16.8–20.4)	1.0
Simmer sauces	17.8 (16.7–18.8)	17.5 (16.8–18.2)	17.7 (16.9–18.5)	0.8	18.4 (16.6–20.3)	18.4 (16.6–20.1)	18.4 (16.6–20.1)	1.0
Other cooking sauces	18.1 (17.2–19.1)	17.6 (17.0–18.3)	18.1 (17.3–18.8)	0.6	19.0 (17.2–20.8)	18.7 (17.1–20.4)	18.9 (17.2–20.6)	0.9

* Adjusted for age, sex, income, education, and working hours.

## Data Availability

Data are available from the investigators upon request, provided all ethical clearances are met.

## Supplementary Materials

The following are available online at https://www.mdpi.com/article/10.3390/nu13051724/s1, Supplementary Methods.
