# Supplementary material for: Correlations between Self-Reported Cooking Confidence and Creativity and Use of Convenience Cooking Products in an Australian Cohort
Source: Nutrients. 2021 May 19;13(5):1724. doi: 10.3390/nu13051724 (PMC8160973; doi:10.3390/nu13051724)
Supplement: Supplementary file 1 [file nutrients-13-01724-s001.zip › nutrients-1189787-supplementary.pdf]

Article

Correlations between self-reported cooking confidence and creativity and use of convenience cooking products in an Australian cohort.

Natasha Brasington [1], Patrice Jones [1,2], Tamara Bucher [1,3] and Emma L Beckett [1,2,3,*]

Supplementary Methods

Cooking confidence scale

	Strongly disagree 1	2	3	4	Strongly agree 5
I am a good cook	○	○	○	○	○
Others view me as a good cook	○	○	○	○	○
I am a relatively better cook than my friends/family	○	○	○	○	○
I get a sense of satisfaction from preparing and cooking meals	○	○	○	○	○
I am confident that whatever I cook will turn out well	○	○	○	○	○
Others don't consider me to be a good cook	○	○	○	○	○
I dont consider myself to be a good cook	○	○	○	○	○

Cooking creativity scale

Please indicate the extent to which you agree or disagree with the following statements.

	Strongly disagree 1	2	3	4	Strongly agree 5
I consider myself to be very creative with food	O	O	O	O	O
I am good at coming up with new and different recipe ideas	O	O	O	O	O
I dont have much of an imagination about things to cook	O	O	O	O	O
People who know me would say that I am more creative with food than most people	O	O	O	O	O
I like thinking of original and new things to cook	O	O	O	O	O
I prefer to follow recipes in cook books	O	O	O	O	O
